# Still reduced cardiovascular mortality 12 years after supplementation with selenium and coenzyme Q10 for four years: A validation of previous 10-year follow-up results of a prospective randomized double-blind placebo-controlled trial in elderly

**DOI:** 10.1371/journal.pone.0193120

**Published:** 2018-04-11

**Authors:** Urban Alehagen, Jan Aaseth, Jan Alexander, Peter Johansson

**Affiliations:** 1 Division of Cardiovascular Medicine, Department of Medical and Health Sciences, Linköping University, Linköping, Sweden; 2 Research Department, Innlandet Hospital Trust and Inland Norway University of Applied Sciences, Elverum, Norway; 3 Norwegian Institute of Public Health, Oslo, Norway; 4 Department of Social and Welfare studies, Linköping University, Norrköping, Sweden; 5 Department of Medical and Health Sciences, Linköping University, Norrköping, Sweden; University of Adelaide, AUSTRALIA

## Abstract

**Background:**

Selenium and coenzyme Q10 are both necessary for optimal cell function in the body. The intake of selenium is low in Europe, and the endogenous production of coenzyme Q10 decreases as age increases. Therefore, an intervention trial using selenium and coenzyme Q10 for four years as a dietary supplement was performed. The main publication reported reduced cardiovascular mortality as a result of the intervention. In the present sub-study the objective was to determine whether reduced cardiovascular (CV) mortality persisted after 12 years, in the supplemented population or in subgroups with diabetes, hypertension, ischemic heart disease or reduced functional capacity due to impaired cardiac function.

**Methods:**

From a rural municipality in Sweden, four hundred forty-three healthy elderly individuals were included. All cardiovascular mortality was registered, and no participant was lost to the follow-up. Based on death certificates and autopsy results, mortality was registered.

**Findings:**

After 12 years a significantly reduced CV mortality could be seen in those supplemented with selenium and coenzyme Q10, with a CV mortality of 28.1% in the active treatment group, and 38.7% in the placebo group. A multivariate Cox regression analysis demonstrated a reduced CV mortality risk in the active treatment group (HR: 0.59; 95%CI 0.42–0.81; P = 0.001). In those with ischemic heart disease, diabetes, hypertension and impaired functional capacity we demonstrated a significantly reduced CV mortality risk.

**Conclusions:**

This is a 12-year follow-up of a group of healthy elderly participants that were supplemented with selenium and coenzyme Q10 for four years. Even after twelve years we observed a significantly reduced risk for CV mortality in this group, as well as in subgroups of patients with diabetes, hypertension, ischemic heart disease or impaired functional capacity. The results thus validate the results obtained in the 10-year evaluation.

The protective action was not confined to the intervention period, but persisted during the follow-up period. The mechanisms behind this effect remain to be fully elucidated, although various effects on cardiac function, oxidative stress, fibrosis and inflammation have previously been identified. Since this was a small study, the observations should be regarded as hypothesis-generating.

**Trial registration:**

Clinicaltrials.gov NCT01443780.

## Introduction

Selenium is a trace element that can be found in all living cells[1, 2]. Important selenoproteins in the body are selenoprotein P, glutathione peroxidases, and thioredoxin reductase, all protecting against oxidative stress. Increased vascular oxidative stress and endothelial dysfunction in patients with coronary heart disease have been reported, although the results are conflicting[3, 4]. In European populations with low dietary selenium intakes as a result of the low selenium content in the soil, biofortification has been regarded as logical[5, 6], as opposed to the status in the United States where the selenium soil content is generally high. The estimated serum selenium concentrations in US citizens are generally above 120 μg/L,[7, 8] whereas concentrations below 90 μg/L are invariably reported from European countries.[9–13].

Xia et al. demonstrated an interrelationship between selenium and coenzyme Q10 (ubiquinone) in the metabolic pathway to the active form of coenzyme Q10 (ubiquinol) [14]. Moreover, an adequate presence of coenzyme Q10 is needed for optimal functioning of selenoproteins. Similarly, a deficiency of selenium could influence the ability to obtain adequate concentrations of active coenzyme Q10 in cellular compartments. Coenzyme Q10 is a powerful anti-oxidant protecting against lipid peroxidation[15]. It has been shown that ubiquinone reduces the inflammatory response [16]. The endogenous production of coenzyme Q10 decreases continually after the age of 20, and the endomyocardial production is reduced to half at the age of 80 [17]. Thus, elderly people living in geographical areas with low selenium content in the soil and food may be at increased risk of heart disease and premature death due to a possible deficiency of these antioxidants. Our research group have recently reported higher CV mortality in a community population with low plasma selenium concentration [18].

We have previously reported on a dietary supplementation trial with both selenium and coenzyme Q10 to 443 elderly Swedish community members performed during 2003 until 2010 [19]. The intervention time was four years, and the follow-up after 5.2 years showed significantly reduced CV mortality, improved cardiac function as evaluated by echocardiography, and a reduced increase of the N-terminal fragment of proBNP (NT-proBNP), a cardiac peptide biomarker. The long-term effects as seen after ten years have also been reported by our group, where we still observed a significant reduction of CV mortality, even if the intervention lasted for only four years[20]. Positive effects could also be seen in some subgroups of the study population.

The primary aim of the present study was to evaluate possible CV effects of the intervention in the same population 12 years after the introduction of a four-year period of supplementation as a way to validate the surprising results from the 10-year evaluation. A secondary aim was to determine whether positive effects on CV risk could also be seen after 12 years in the two genders, in those with diabetes, ischemic heart disease (IHD), hypertension and impaired functional capacity as measured by the New York Heart Association functional Class (NYHA class).

## Materials and methods

The design of the main study has been published elsewhere [19]. In brief, 443 elderly healthy participants were given dietary supplementation of 200 mg/day of coenzyme Q10 capsules (Bio-Quinon 100 mg B.I.D, Pharma Nord, Vejle, Denmark) and 200 μg/day of organic selenium yeast tablets (SelenoPrecise 200 μg, Pharma Nord, Vejle, Denmark), or a similar placebo during 48 months, and then the interventions was finished. The study supplementation was taken in addition to regular medication if used. All study medications (active drug and placebo) not consumed were returned and counted. All participants were examined by one of three experienced cardiologists. A new clinical history was recorded, and a clinical examination was performed, including blood pressure, assessment of New York Heart Association functional class (NYHA class) as well as ECG and echocardiography. Doppler echocardiographical examinations were performed with the participant in the left lateral position. The ejection fraction (EF) readings were categorized into four classes with interclass limits placed at 30%, 40% and 50% [21, 22]. Normal systolic function was defined as EF≥ 50%, while severely impaired systolic function was defined as EF< 30%. The first participant was included in January 2003, and the last participant concluded the study in February 2010.

**Fig 1:**
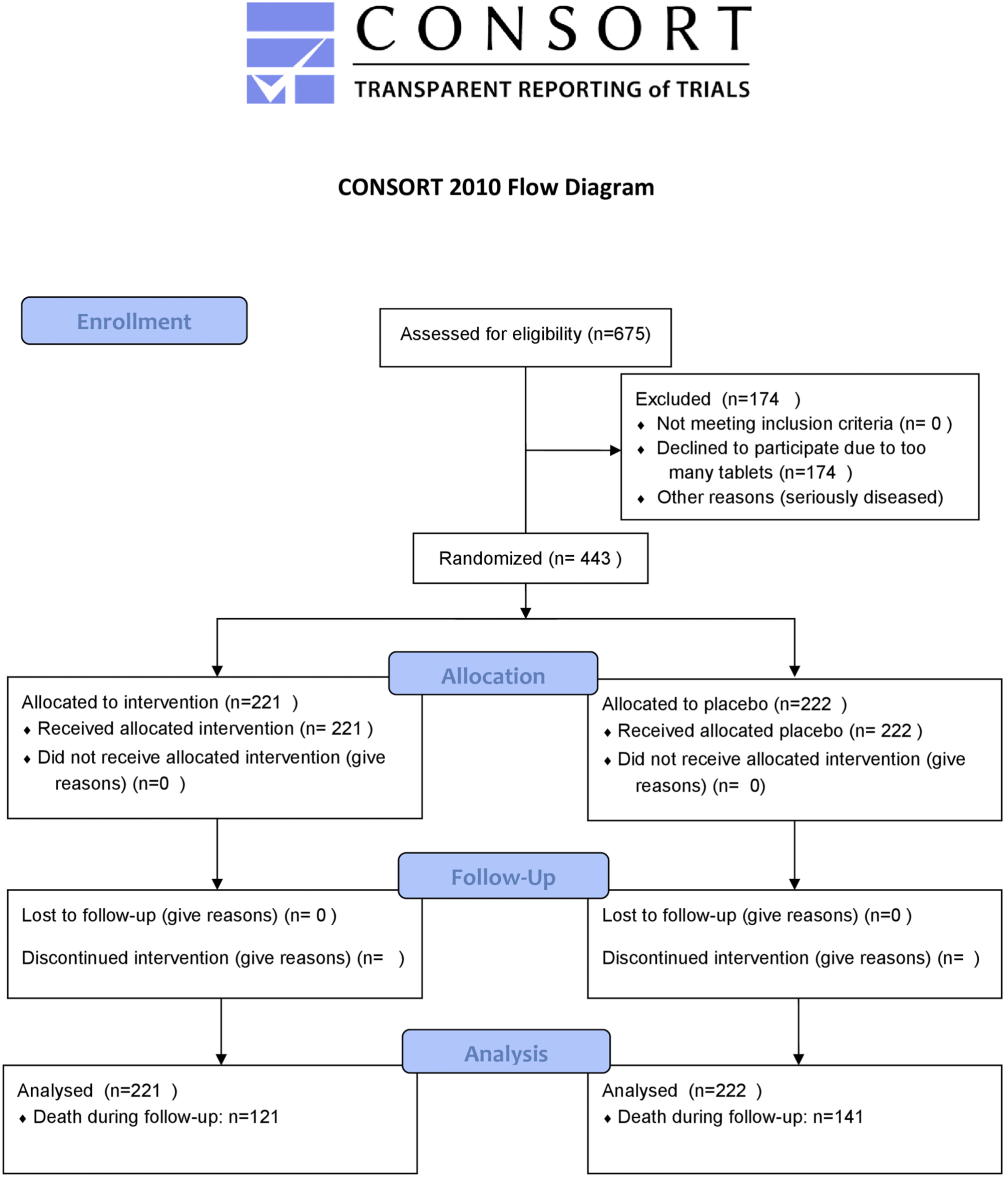
CONSORT flow chart of the study.

As the intervention time was unusually long for the main intervention study (48 months) only 228 participants completed the study; 86 died during the intervention time, and 129 (29.1%) decided not to complete the study. The reasons for the latter have been presented in detail in the main publication, but the main reason was there were too many tablets to take [19]. A flowchart of the total follow-up period is presented as Fig 1.

Written, informed consent was obtained from all patients. The study was approved by the Regional Ethical Committee in Linköping, Sweden and conforms to the ethical guidelines of the 1975 Declaration of Helsinki. This study was registered at Clinicaltrials.gov, and has the identifier NCT01443780. As the trial started when registration in Clinicaltrials.gov, or elsewhere was optional, the trial was not registered at the start. Registration took place at the time of publication, when this became obligatory. There is presently no ongoing study from the research group with the described preparations.

### Biochemical analyses

All blood samples were obtained while the patients were at rest in a supine position. The blood samples were collected in plastic vials containing EDTA (ethylenediamine tetracetic acid). The vials were placed on ice before chilled centrifugation at 3000g, and then frozen at -70 °C. No sample was thawed more than twice.

### Mortality

All instances of cardiovascular mortality (CV mortality) were registered. The mortality information was obtained from the National Board of Health and Welfare in Sweden, which registers all deaths of Swedish citizens based on death certificates or autopsy reports. Written, informed consent was obtained from all patients.

### Statistical methods

Descriptive data are presented as percentages. Chi-square tests were used for discrete variables. All evaluations are performed according to the intention-to-treat principle. Kaplan-Meier analysis was used to demonstrate CV mortality during the follow-up period. Cox proportional hazard regression analysis was used to evaluate risk of CV mortality. The independent variables included in the multivariate model were variables known to be associated with CV mortality: age, male gender, smoking, hypertension, diabetes, IHD, NYHA class III, treatment with beta blocker, ACE-inhibitor, Hb<120g/L, EF<40% and active treatment with selenium and coenzyme Q10.

A P-value of <0.05 was considered statistically significant. All data were analyzed using standard software (Statistica v. 13.2, Dell Inc, Tulsa, OK).

## Results

The study population were followed regarding CV mortality during a median follow-up time of 4233 days (range 348–5275), i.e. about 12 years. In the survival group the median follow-up time was 4484 days (range 404–5275), and in the CV mortality group a median follow-up time of 2818 days (range 348–4869) was recorded.

From the basal characteristics it could be seen that the two populations were balanced at the start of the intervention in all but one variable, use of ACE-inhibitors (15.8% vs 24.3%; *P* = 0.03). After 12 years there was no significant difference between the basal characteristics variables (Table 1).

**Table 1:** Population characteristics at inclusion, and after 12 years.

	At study start			After 12 years		
	Active	p-value	Placebo	Active	p-value	Placebo
**n**	221		222	100		81
**Age, mean**	78		78	75		76
**Males/Females, n**	115/106		110/112	42/58		33/48
**Smokers, n (%)**	21 (9.5)	0.86	20 (9.0)	2 (2.0)	0.27	4 (4.9)
**Diabetes, n (%)**	47 (21.3)	0.95	48 (21.6)	19 (19.0)	0.60	13 (16.1)
**Hypertension, n (%)**	158 (71.5)	0.28	168 (75.7)	69 (69.0)	0.84	57 (70.4)
**IHD, n (%)**	47 (21.3)	0.51	53 (23.9)	13 (13.0)	0.35	7 (8.6)
**NYHA class III, n (%)**	41 (18.6)	0.49	47 (21.2)	12 (12)	0.46	7 (8.6)
**Medical Treatment**						
**ACEI, n (%)**	35 (15.8)	0.03	54 (24.3)	10 (10.0)	0.32	12 (14.8)
**Beta blockers, n (%)**	81 (36.7)	0.40	73 (32.9)	37 (37.0)	0.11	21 (25.9)
**Statins, n (%)**	45 (20.7)	0.50	51 (23.0)	20 (20.0)	0.97	16 (19.8)
**Examinations**						
**Hb<120g/L, n (%)**	23 (10.4)	0.39	29 (13.1)	10 (10.0)	0.98	8 (9.9)
**EF<40%, n (%)**	16 (7.2)	0.87	17 (7.7)	5 (5.0)	0.98	4 (4.9)

Note: ACEI: ACE- inhibitors; EF: Ejection fraction according to echocardiography; IHD: Ischemic heart disease; NYHA: New York Heart Association functional class

Upon analyses of the basal characteristics, it could be seen that at the start of the intervention the active treatment group, and the placebo group had the same age (78 years), whereas in survivors after 12 years the mean age had decreased to 75 years in the active treatment group, and to 76 years in the placebo group. At the start of the intervention equal proportions of the two groups had diagnosed diabetes (21%), whereas after 12 years a reduction of the diabetic proportion could be seen in both groups, though it was more prominent in the placebo group (Table 1). The numbers with diagnosed hypertension were equal both in the groups at the start of the intervention, and after 12 years. Regarding IHD, the intervention and placebo groups were balanced both at the start and after 12 years; however, both groups exhibited a decrease in the number with IHD after 12 years.

### Cardiovascular mortality within 12 years

**Fig 2:**
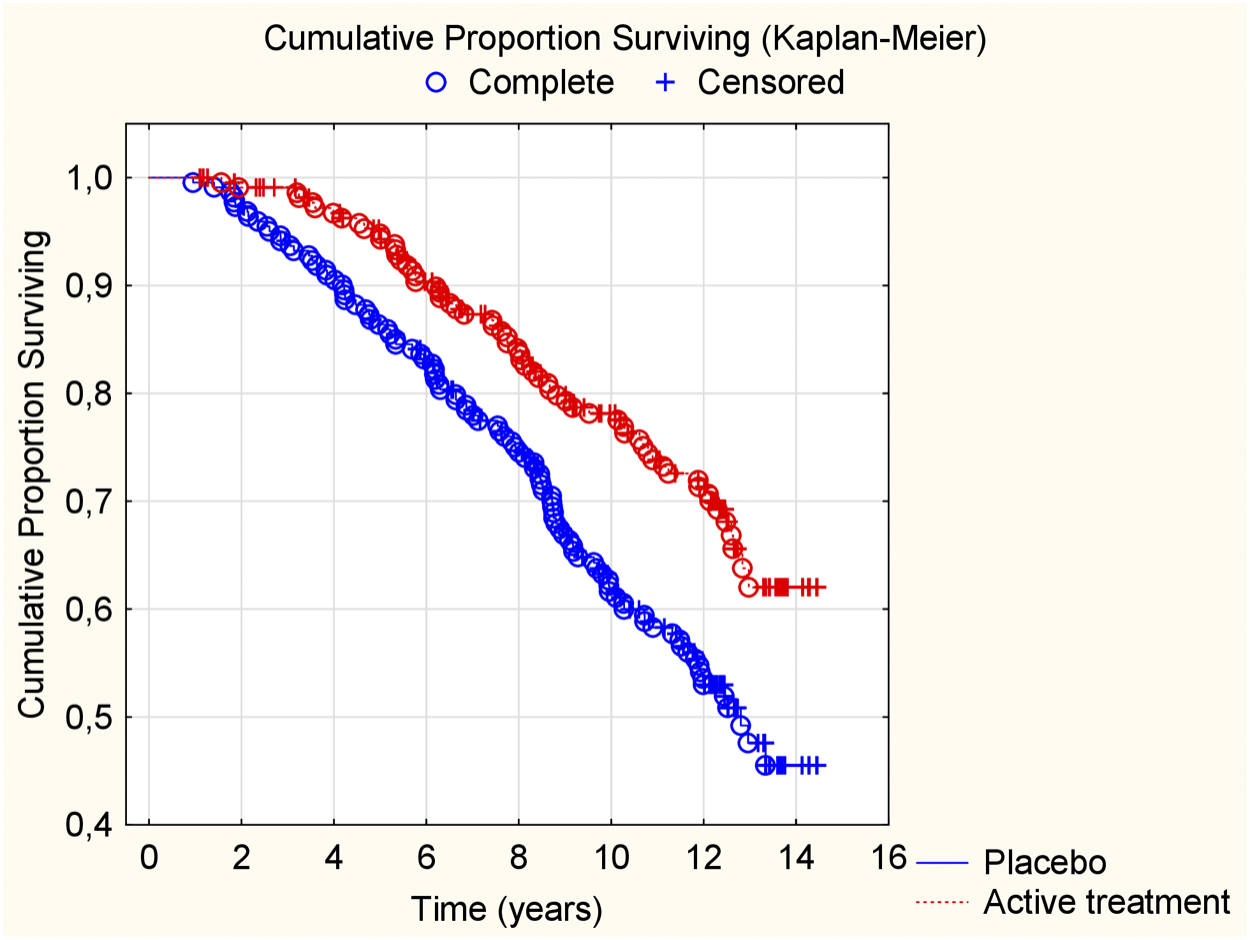
Kaplan-Meier graph illustrating cardiovascular mortality during a follow-up period of 12 years of those supplemented with selenium and coenzyme Q10 versus placebo for four years on top of regular pharmaceutical treatment.

When evaluating the CV mortality within the 12-year period, we found that those on active treatment during the intervention had a significantly lower mortality also after 12 years (active treatment group: 62/221; 28.1%, vs placebo: 86/222; 38.7%; *χ*^*2*^:13.8; *P*<0.0001). The differences in CV mortality during the follow-up were also assessed and displayed in a Kaplan-Meier graph, showing a clear separation of the two groups (Fig 2).

When applying the CV mortality results in univariate Cox regressions, we observed a highly significant risk reduction resulting from supplementing the participants with selenium and coenzyme Q10 for four years, also after 12 years (HR:0.58; 95%CI: 0.42–0.79; P<0.0007). Applying the data into a multivariate Cox regression model where well-known variables influencing the cardiovascular risk were included, a cardiovascular risk reduction appeared to remain also after 12 years (HR:0.59; 95%CI: 0.42–0.81: P = 0.001) (Table 2).

**Table 2:** Cox proportional hazard regression analysis evaluating risk of cardiovascular mortality by supplementation of selenium and coenzyme Q10 combined in a multivariate model after 12 years of follow-up after 4 years of intervention to an elderly community population.

Variables	Hazard ratio	95%CI	*P*-value
**Age**	1.16	1.11–1.22	<0.0001
**Male gender**	1.80	1.30–2.51	<0.0001
**Smoking**	1.71	1.08–2.71	0.02
**Hypertension**	1.23	0.85–1.78	0.27
**Diabetes**	1.30	0.92–1.86	0.14
**IHD**	1.50	1.02–2.21	0.04
**NYHA class 3**	2.01	1.45–3.03	<0.0001
**Beta blocker**	0.85	0.59–1.21	0.37
**ACE-inhibitor**	1.11	0.76–1.61	0.59
**Hb<120g/L**	1.04	0.66–1.65	0.85
**EF<40%**	0.88	0.50–1.55	0.66
**Active treatment**	0.59	0.42–0.81	0.001

Notes: EF: Ejection fraction; IHD: Ischemic heart disease; NYHA: New York Heart Association functional class; Q4: 4^th^ quartile

### Subgroup analyses

When analyzing the two genders separately, we found a highly significant difference in CV mortality between the female intervention and the placebo groups, 20/106, and 46/112, respectively (*χ*^2^:12.7; *P* = 0.0004) after up to 12 years of follow-up. Similarly, in the male group a trend for difference between the groups was found (active group: 42/115, compared with the placebo group; 54/110; *χ*^2^:3.63; *P* = 0.057).

**Fig 3:**
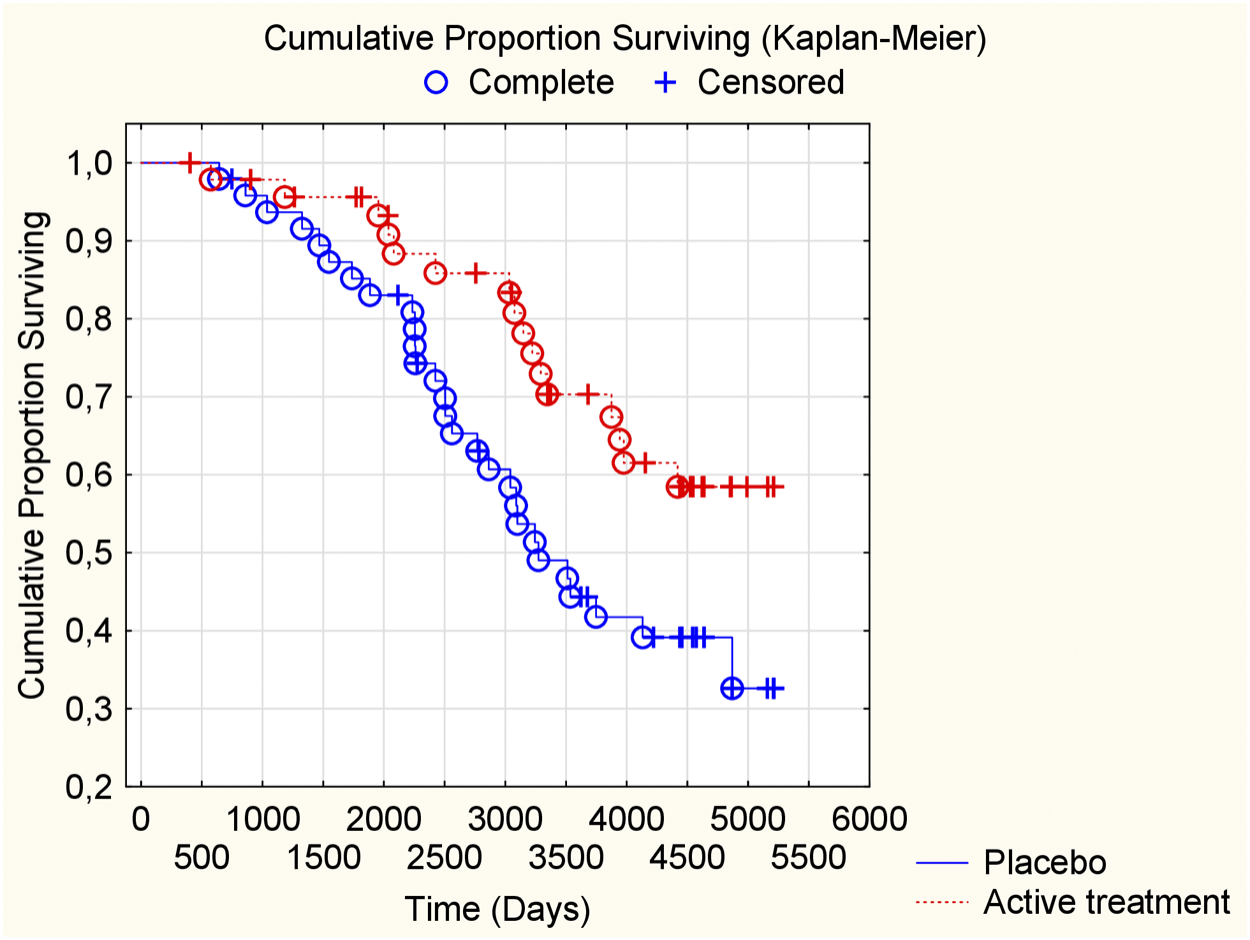
Kaplan-Meier graph illustrating cardiovascular mortality in participants with ischemic heart disease during a follow-up period of 12 years of those supplemented with selenium and coenzyme Q10 versus placebo for four years on top of regular pharmaceutical treatment.

In the group with IHD we found a reduced CV mortality risk after supplementation with selenium and coenzyme Q10, also after 12 years of follow-up, in comparison with those on placebo (HR: 0.52; 95%CI: 0.30–0.90; *P* = 0.02) (Fig 3).

**Fig 4:**
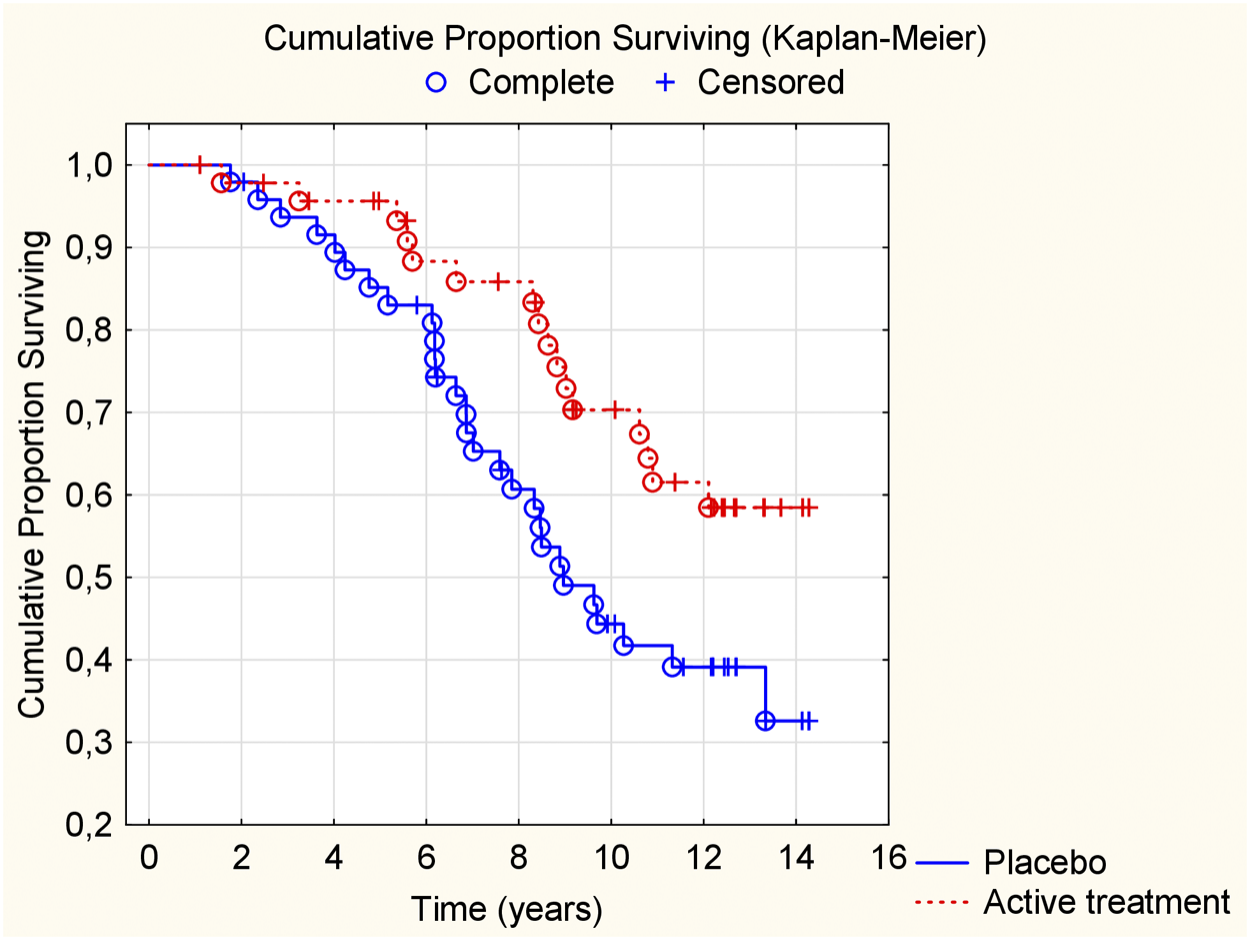
Kaplan-Meier graph illustrating cardiovascular mortality in participants with diabetes during a follow-up period of 12 years of those supplemented with selenium and coenzyme Q10 versus placebo for four years on top of regular pharmaceutical treatment.

For the group with diabetes we also observed a reduced CV mortality risk after 12 years in the active supplementation group (HR: 0.50; 95%CI: 0.27–0.93; *P* = 0.03) (Fig 4).

**Fig 5:**
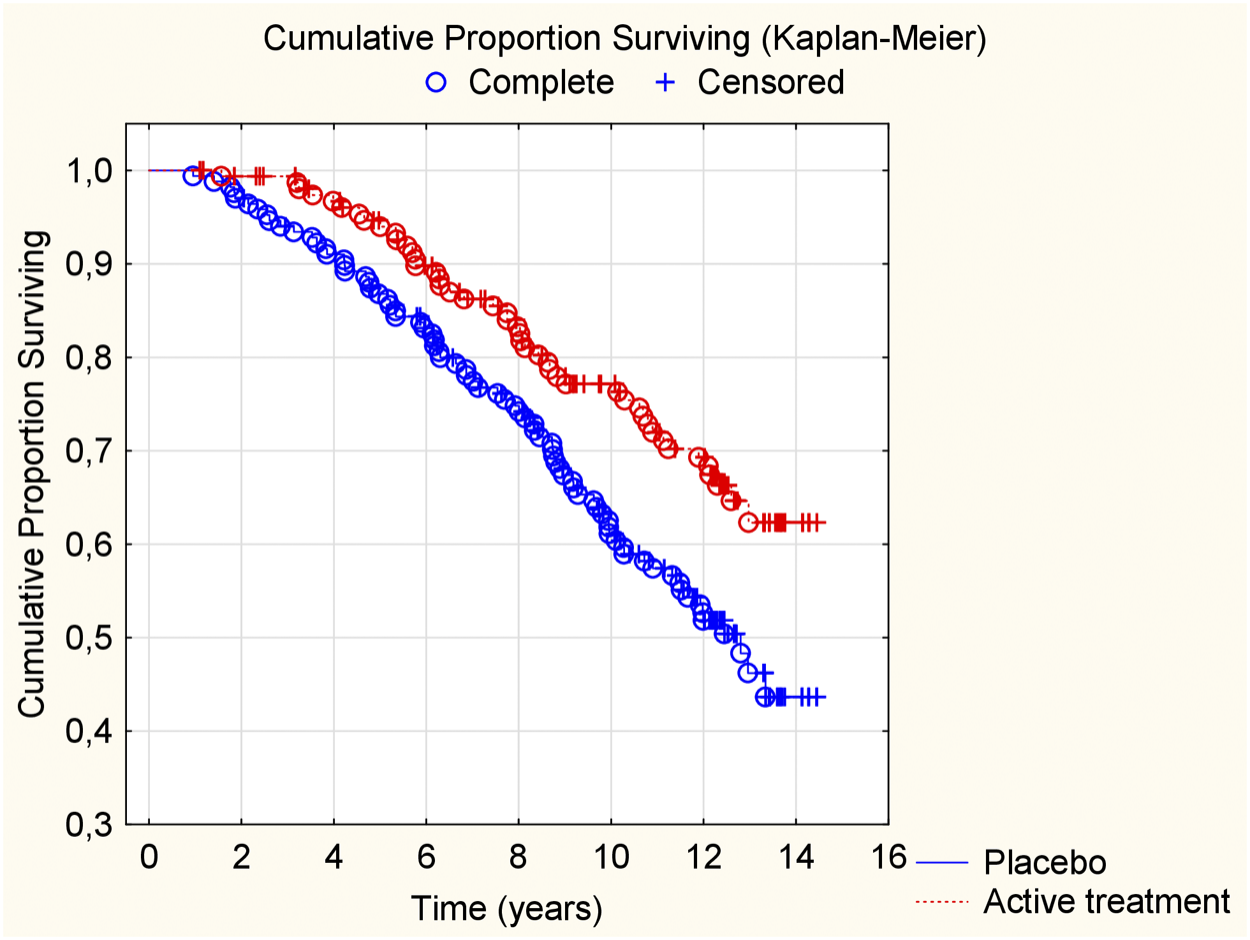
Kaplan-Meier graph illustrating cardiovascular mortality in participants with hypertension during a follow-up period of 12 years of those supplemented with selenium and coenzyme Q10 versus placebo for four years on top of regular pharmaceutical treatment.

In the group with hypertension, a reduced CV mortality risk reduction could be seen as well (HR: 0.60; 95%CI: 0.41–0.85; *P* = 0.005) (Fig 5).

**Fig 6:**
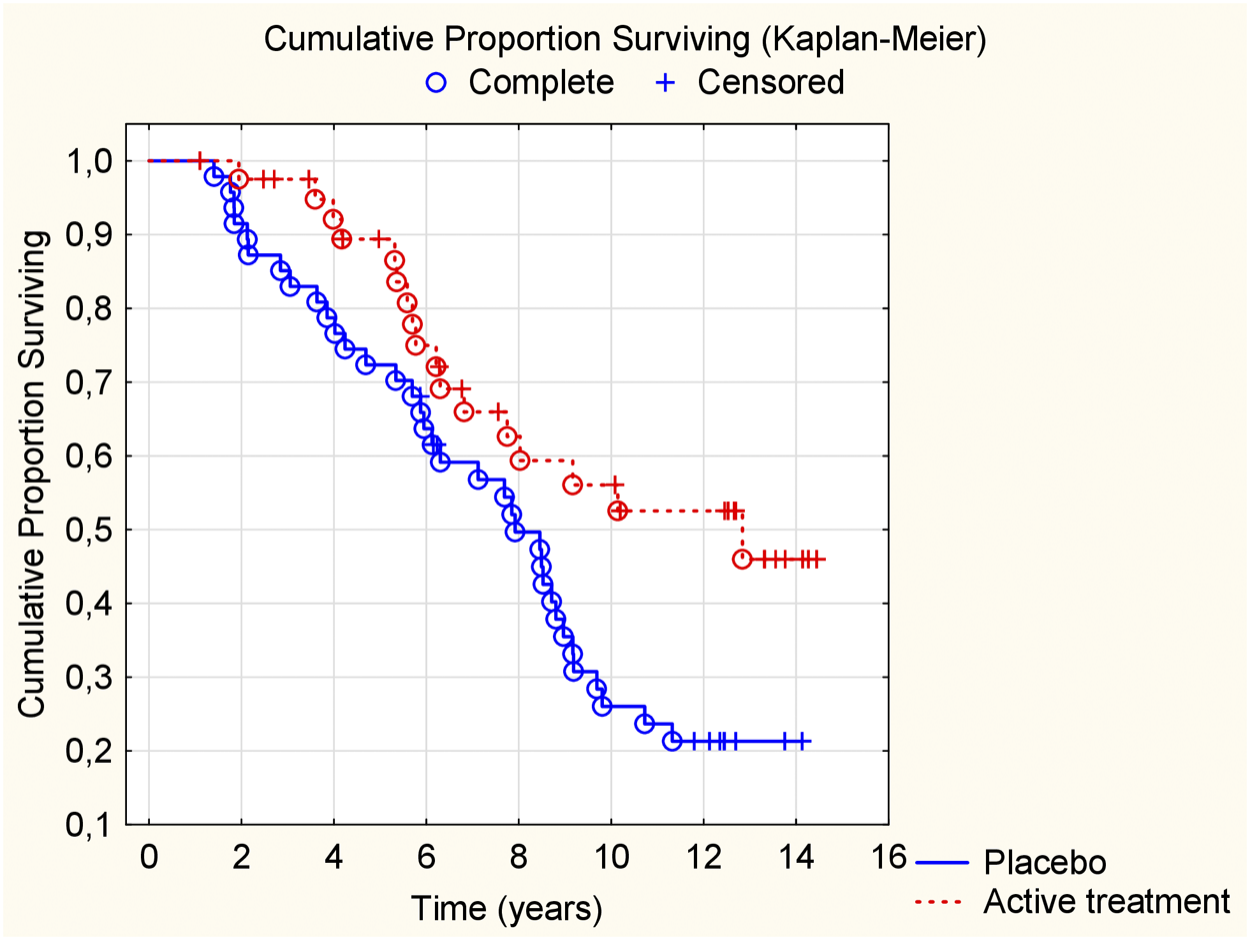
Kaplan-Meier graph illustrating cardiovascular mortality in participants with NYHA functional class III during a follow-up period of 12 years of those supplemented with selenium and coenzyme Q10 versus placebo for four years on top of regular pharmaceutical treatment.

For those with a severe functional impairment due to reduced cardiac function (NYHA functional class III) a significant reduction of CV mortality risk could be observed (HR: 0.49; 95%CI: 0.27–0.88; *P* = 0.002) (Fig 6).

If combining those with IHD and those with impaired systolic cardiac function (EF<40%), a reduced risk for CV death within the 12-year period could be found with an HR of 0.56 (95%CI: 0.33–0.05; *P* = 0.03) (Table 3).

**Table 3:** Model testing the effect of intervention after 12 years on risk for cardiovascular mortality in different groups.

Variable	HR	p-Value	95%CV
**Total study population**	0.58	0.0007	0.42–0.70
**HT**	0.59	0.005	0.41–0.85
**IHD**	0.52	0.02	0.30–0.90
**DM**	0.50	0.03	0.27–0.93
**NYHA III**	0.49	0.02	0.27–0.88
**IHD+EF<40%**	0.56	0.03	0.33–0.95
**IHD+EF<40%+HT**	0.60	0.004	0.42–0.84
**IHD+EF<40%+HT+DM**	0.59	0.003	0.42–0.83

Note: CV: Coefficient of variation; DM: Diabetes; HT: Hypertension; HR: Hazard ratio; IHD: Ischemic heart disease; NYHA III: New York Heart Association functional class III

In order to further increase the group at CV risk, we combined the groups with hypertension, IHD and impaired systolic function. In this group, we found a significant risk reduction with an HR of 0.60 (95%CI: 0.42–0.84; *P* = 0.004) as a result of the intervention (Table 3).

By adding those with diabetes to the above group, a significant risk reduction was still observed, with an HR of 0.59 (95%CI: 0.42–0.83; *P* = 0.003)(Table 3).

## Discussion

In an intervention study in 443 elderly healthy persons, selenium and coenzyme Q10 were given as a combined dietary supplement for four years. After this period no intervention was given, and thus some of the participants have been without the selenium and coenzyme Q10 intervention for ten years. Our results show a continual and significant reduction in CV mortality during the whole follow-up period of 12 years, which also included the eight-year period after termination of the intervention (Table 4).

**Table 4:** Difference in cardiovascular mortality within 5, 10 and 12 years after intervention of selenium and coenzyme Q10 combined or placebo for four years.

Follow-up time	Mortality in active treatment group (%)	Mortality in placebo group (%)	*χ*^2^-value	P-value
**5 years**	5.9	12.6	5.97	0.015
**10 years**	20.8	38.7	17.01	<0.0001
**12 years**	28.1	45.0	13.78	0.0002

This appears clearly from the calculated mortality rates of the active treatment group, in comparison with those of the placebo group (Table 5), as we also did in the 10 years evaluation [20].

**Table 5:** Mortality rate after 5 years, 10 years and 12 years in the active treatment group compared to the placebo group, and to official mortality statistics.

	5.2 years of follow-up		10 years of follow-up		12 years of follow-up	
	All-cause mort rate	Cardiovasc mort rate	All-cause mort rate	Cardiovasc mort rate	All-cause mort rate	Cardiovasc mort rate
**Active group**	2433	1130	4427	2079	4542	2327
**Placebo group**	3115	2423	5400	3870	5993	3754
**Reference pop**	5794	2144	15,241*	6998*	15,420**	6326**

Note: Mortality rate expressed as mortality /100,000/year

Note: Reference group: Official Swedish mortality statistics based on the age group 80–84 in the 5.2 year follow-up, and on the age group 85 years and above in the 10 years follow-up;

* Figures based on statistics from 2014;

** Figures based on statistics from 2016

In Table 5, the figures from official Swedish mortality statistics are also added for comparison. The sample size of the present study is relatively small; therefore, the figures should be interpreted with caution. However, it is striking that the reduction in CV mortality also remains after 12 years. Even though the reduction in CV risk measured as reduced CV mortality is still significant, the effect seems somewhat less after 12 years than after ten years [21], especially in the male group.

This can probably be explained by the fact that ever-existing pathogenic factors catch up with the positive effects obtained by our intervention. Also, as reported previously, females have lower levels of coenzyme Q10 than males, presumably explaining why the females benefited more than males from the supplementation [23].

The CV mortality risk reduction is significant and stable in all the well-known risk populations, including those with IHD, hypertension or diabetes. Even after combining groups in order to increase stepwise the size to the subpopulations, the risk reduction is stable and comparable to the risk reduction obtained in the separate subgroups, indicating a robust effect caused by the intervention. It is tempting to speculate that permanent or progressive structural changes took place in the subjects of the placebo group during the interventional four-year period, explaining the apparent slowing down of CV pathogenesis in the supplemented group.

Selenium-containing enzymes, as well as the coenzyme ubiquinone are strong antioxidants, both being required in adequate amounts for normal cell function. Positive effects of supplementation of coenzyme Q10 on endothelial function have also been reported [24]. It has been shown that increased inflammatory activity[25–27], and oxidative stress occur in the elderly [28], and as one of the effects of the present intervention is to reduce signs of inflammatory activity [29], and oxidative stress [30], it is tempting to speculate that this effect is partially responsible for the long-lasting protection obtained in the present study. Moreover, endothelial cells and platelets may be particularly vulnerable to oxidative stress as they are surrounded by continuous oxygen transport in the circulation, and also exposed to increasing inflammatory burden with age [31, 32].

Furthermore, the persisting effects even eight years after termination of the supplementation could be related to the unusually long intervention time of four years, which as discussed above, presumably was paralleled by structural changes in the placebos. The mean plasma concentration in this population was 67 μg/L[18]. For full expression of the extracellular selenoprotein P, a concentration of >90–140 μg/L is needed [33–35], implying that a selenium deficiency in fact existed before the intervention and during the whole period in the placebo group. Apparently, the size of the intervention, 200 μg Se/day was enough to give the needed blood concentration, which has been reported previously [36]. On average, the supplemented group had an estimated total daily selenium intake of about 235 μg, which is well above the requirement to optimize selenoprotein P. It was also below a tolerable upper intake level of 300 μg as recommended by the European Food Safety Authority [37] and Nordic Nutrition Recommendations [38]. In contrast, the population studied in the American SELECT trial had a basal Se intake of at least 120 μg/d before supplementation; thus, their supplementation of 200 μg Se/d brought them into a marginal or hazardous zone of above 300 μg [39, 40].

The long-lasting effect of the supplementation might also be related to the fact that a major component of yeast selenium is selenomethionine, which is, at the expense of methionine, incorporated into non-seleno proteins, which constitute the unspecific selenium pool having a long elimination half-life [41]. Another partial explanation of the long-lasting effects might be that the municipality under study developed an increased interest in supplementation with selenium and coenzyme Q10 after the intervention period, and therefore some of the participants of the project may have continued to take supplementation. However, if this was the case, even though it was not investigated in this study, we would expect that the amount randomized to the placebo group that might have started post-project supplementation by random be approximately as those that previously belonged to the active treatment group, and as a result of the post-project supplementation, the difference between the groups should disappear.

The present 12-year evaluation of cardiovascular mortality after four years of intervention is unique, and should be regarded as a validation of the surprising results from the 10-year evaluation, and shows that the positive effects of the intervention persist.

Therefore, the hypothesis arising from our results remains that the intervention with selenium and coenzyme Q10 inhibits the pathogenesis of irreversible, presumably structural, changes preceding cardiovascular events.

## Limitations

The presented study has a limited sample size, making the interpretation of the results difficult. However, the statistical evaluations are extensive due to the many evaluations performed in the study population, including those previously published.

The evaluations of the subgroups are even more uncertain as the sample sizes are smaller compared to the main study group. However, also in this respect we would like to argue that the different types of statistical methods used all point in the same direction; a reduction of mortality was obtained by the intervention, confirming the previous suggestions that the optimal selenium intake lies in the range 100–300 μg/d.

In this report the CV mortality within 12 years has been analyzed, a variable that could be uncertain as it was based mainly on death certificates, and not on autopsy in a majority of the participants. However, it is likely that the uncertainties in this study are of the same extent as previous mortality studies that are not based on autopsies only. Hence, in spite of the limitation we think our results provide interesting information.

The limited age span of the analyzed study population also represents a limitation, making extrapolation of the results to larger populations difficult. However, the fact that the incidences of CV as well as of other types of disease are higher in an elderly population compared to younger persons makes the obtained results of this 12-year follow-up even more intriguing.

## Conclusion

We present a 12-year analysis of CV mortality in an elderly Swedish population that had been given supplementation with selenium and coenzyme Q10 as a contribution to their diet for four years. The present follow-up revealed a reduced CV mortality risk of more than 40%, and a significant risk reduction in those with hypertension, IHD, impaired cardiac function, and diabetes. We consider that the presented data, based on small sample sizes, should be regarded as hypothesis-generating, as the data are both intriguing and surprising.

## Supporting information

S1 Study Protocol (DOCX)

S1 Consort Checklist (DOC)
